# Level-1-visual perspective taking for human and robot avatars

**DOI:** 10.1007/s00426-025-02188-z

**Published:** 2025-11-19

**Authors:** Christine Blech, Hanna Lembcke, Cédric A. Bouquet, Roman Liepelt

**Affiliations:** 1 Department of General Psychology: Judgment, Decision Making, Action, Faculty of Psychology, FernUniversität in Hagen, Hagen, Germany; 2 Université Clermont Auvergne, CNRS, LAPSCO, Clermont-Ferrand, France

**Keywords:** Level 1 visual perspective taking, Human robot interaction, Robots, Avatars, Anthropomorphism

## Abstract

Research on level 1 visual perspective taking (L1-VPT) has been debating whether L1-VPT is an implicit socially rooted or rather a non-social process. Using online versions of the Dot Perspective Task by Samson et al. (*Journal of Experimental Psychology: Human Perception and Performance, 36*(5), 1255–1266, 2010) we approached this question by comparing L1-VPT for robot vs. human avatars. In line with the assumption that visual perspective taking is due to mentalizing, we predicted that perspective taking, leading to altercentric intrusions, should occur more strongly for the human avatars than for the robot avatars. In two experiments, a within-participant design was applied: 2 (avatar: human vs. robot) × 2 (avatar perspective: consistent vs. inconsistent) × 2 (task: avatar perspective vs. self-perspective). The human avatar was a male in Experiment 1 (*n* = 120) and a female in Experiment 2 (*n* = 113). The analyses of reaction times and error rates showed significant, medium to large egocentric intrusions and significant, small to medium altercentric intrusions for both avatar types, suggesting interference from the irrelevant perspective. Against the prediction, the altercentric intrusions for human avatars were not significantly larger than for robot avatars. Taking into account methodological concerns and suggesting future experimental variations, we argue that the submentalizing approach assuming that visual perspective taking is based on domain general processes provides a good explanation for our results.

Understanding what another person thinks, plans, knows, or believes is crucial for successful social interactions in our daily lives. As early as 2–3 years of age, and possibly even before, young children begin to develop a *Theory of Mind* (Premack & Woodruff, 1978) that becomes more differentiated and refined over the years (Rakoczy, 2022). One pre-requisite for the Theory of Mind is *perspective taking*, a concept that can broadly be divided into three dimensions (Kurdek & Rodgon, 1975; see also Erle & Topolinski, 2017): cognitive (knowledge and intentions), affective (emotional states) and perceptual (viewpoint). Relating to the perceptual dimension, visual perspective taking, i.e., the ability to form a representation of another’s visual experience or to perceptually simulate the image seen from another person’s point of view (Samuel et al., 2021), has been subdivided into two related, but distinct levels (Flavell et al., 1981): Level 1 visual perspective taking (L1-VPT) refers to the ability to know *what* another agent is seeing or not seeing; Level 2 visual perspective taking (L2-VPT) concerns the ability to know *how* another agent is seeing an object. The latter requires not only visual, but also spatial abilities (i.e., mental rotation), but seems to rely on the same initial processing strategies as L1-VPT (Surtees et al., 2013).

While there has already been much research on visual perspective taking of human agents and human interaction partners (for an overview see Holland et al., 2021; Samuel et al., 2023), the extended use of robots, not only in traditional industrial settings, but also in the service and health sector, makes the social aspects of human-robot interactions increasingly relevant (Duffy et al., 2003; Naneva et al., 2020). In our study, we will combine this applied context of human-robot interaction with one basic subcomponent of perspective taking: L1-VPT. Several studies have investigated L1-VPT using an experimentally controlled response time task, the *Dot Perspective Task* (Samson et al., 2010). In this task, participants are shown images of a room and asked to count dots appearing on the walls, either from their own perspective or from the perspective of an avatar placed in the middle of the displayed room. The two perspectives can either be *consistent*, when the number of dots seen by the avatar is equal to the total number of dots in the room (as seen by participants), or *inconsistent*, when participants, from their perspective, see more dots than the avatar (with some dots on the wall behind the avatar).

Many authors that have conducted experiments with different types of avatars and varying degrees of perspective saliency within the Dot Perspective Task found evidence for *egocentric intrusions* (Ferguson et al., 2018; Samson et al., 2010; Xiao et al., 2022): When instructed to adopt the avatar’s perspective, participants react more slowly and make more errors in trials with inconsistent perspectives, as compared to trials with consistent perspectives. Instead of fully inhibiting their own, salient, but here irrelevant perspective, participants seem to automatically process the number of dots from their own perspective before selecting their answer (Qureshi et al., 2010).

Research on *altercentric intrusions* has been somewhat more controversial. Altercentric intrusions occur when the perspective of the avatar is inconsistent with that of participants and interferes with participants’ response. That is, when participants have to respond according to their own perspective, they react more slowly and make more errors on inconsistent vs. consistent trials. This would be indicative that the actually irrelevant perspective of the avatar affects the processing and/or selection of the answer. The most far-reaching interpretation of this finding is the *mentalizing* approach claiming that observers automatically and spontaneously process the avatar’s perspective (Furlanetto et al., 2016; Frith & Frith, 2012; Qureshi et al., 2010; Ramsey et al., 2013; Samson et al., 2010; Surtees & Apperly, 2012; Todd et al., 2017). According to Apperly and Butterfill (2009), humans possess an implicit and automatic mentalization system from an early age; as they grow more mature, an additional, explicit system allowing to infer mental states from others develops, but the primary implicit system still persists.

In contrast to the mentalizing approach, other authors have argued that the consistency effects in the Dot Perspective Task arise from non-social, domain-general attentional mechanisms (Cole et al., 2016; Gardner & Thorn, 2025; Heyes, 2014; Santiesteban et al., 2014, 2017), which is sometimes referred to as *submentalizing* (Heyes, 2014). A series of studies contrasted these two accounts (mentalizing vs. submentalizing), for example by using human-like avatars vs. non-social stimuli (e.g., arrows; Santiesteban et al., 2014) or by using avatars who were able to see vs. avatars whose vision was disrupted (e.g., by goggles, see Conway et al., 2017). The results suggest that a combination of domain-specific, social processes and domain-general, non-social processes play a role during different stages of perspective taking, though their influences are not easily disentangled (e.g., Michael et al., 2018; Qureshi et al., 2020).

Acknowledging that there is at least partial support for implicit mentalizing in visual perspective taking, the question arises as to which attributes of an avatar and an observer allow for effective perspective taking. Working memory capacity (cf. Qureshi & Monk, 2018), age (e.g., De Lillo & Ferguson, 2023), as well as social abilities of the observer (e.g., empathy; Kragh Nielsen et al., 2015) are factors, among others, which have already been identified as modulating visual perspective taking. Beyond such characteristics of the observer alone, however, it is important to consider how the characteristics of the observer and the observed agent match.

Research based on the theoretical approach of common coding between perception and action (Dolk et al., 2014; Hommel et al., 2001; Prinz, 1997) suggests that in the context of two acting individuals (joint action), *self-other integration* is typically strengthened when two actors share many features. Transferred to visual perspective taking, enhanced self-other integration due to greater (perceived) similarity between the observing agent (the participant in an experiment) and the observed agent (the avatar in the Dot Perspective Task) could entail stronger automatic perspective-taking. In support of this logic, Ferguson et al. (2018) found that altercentric intrusions, which are considered as indicative of spontaneous perspective taking, were higher when participants and avatars were of the same age (i.e., adult participant and adult avatar) as compared to incongruent ages (i.e., adult participant and child avatar). Paralleling effects involving human vs. humanoid robot avatars have been reported by Xiao et al. (2022): In their second experiment, in a sample of young Chinese university students, the authors found evidence for altercentric intrusions when human participants saw human avatar stimuli, but not when they saw robot avatars. In other words, the similarity between avatar and observer in terms of human-likeness facilitated perspective taking. Zhao and Malle (2022) as well as Wahn et al. (2025) argue in favor of the *mere-appearance hypothesis*, according to which the human or human-like appearance is a sufficient factor to trigger similarity-induced visual perspective taking, whereas beliefs about the avatar (e.g., the avatar’s visual abilities, see Furlanetto et al., 2016) have less impact.

Taking these still relatively few empirical studies on robot avatars in L1-VPT and the self-other integration approach related to the common coding framework (Dolk et al., 2014; Hommel et al., 2001; Prinz, 1997) as a starting point, we tried to replicate the basic experimental design by Xiao et al. (2022) in an online experiment with a European (German speaking) sample of a broader age range, but using a more mechanical, non-humanoid robot. The rationale for choosing a less human-like robot was (a) to maximize the difference between the human vs. robot avatar, and (b) to apply a robot avatar with a higher similarity to robots in everyday contexts like e.g. the service or health sector. Based on the findings of Xiao et al. (2022) and in line with the assumption that visual perspective taking is due to mentalizing, we hypothesized that (non-humanoid) robot avatars do not automatically trigger level 1 visual perspective taking in the Dot Perspective Task to the same extent as human avatars do. We thus predicted that in the Dot Perspective Task the intrusion effects, i.e., (a) the prolonged average response times and/or (b) the increased percent error rates for inconsistent trials as compared to consistent trials, would be higher for human than for robotic avatars. Our original preregistered hypotheses (https://osf.io/zx3wq) addressed not only altercentric, but also egocentric intrusions, both being indicative of interference between self and other perspective. Thus, we will report and discuss both effects, yet with a stronger focus on altercentric intrusions, since altercentrism represents automatic perspective taking its stricter sense.

## Experiment 1

### Method

#### *Sample size rationale*

The targeted sample size was calculated based on an a priori power analysis conducted with PANGEA (*Power ANalysis for GEneral Anova designs*; Westfall, 2016). Xiao et al. (2022) found altercentric and egocentric intrusion effects varying in size from η_p_^2^ = .01 to η_p_^2^ = .44. Hence, also relatively small effect sizes were of interest in the study at hand. Taking a Cohen’s *d* of 0.30 as a starting point, a sample of *N* = 100 was determined to be sufficient to detect intrusion effects in a 2 (consistency) × 2 (avatar) within-subjects interaction in the design outlined below, with a power of .85 and an alpha level of .05. In order to compensate for expected dropouts, and in line with our pre-registration, we aimed for a total of 130 complete records.

#### *Sample*

The data collection took place online from 19 July to 23 August 2023. It was carried out using the survey software Unipark (Tivian XI GmbH, 2022) and the experimental software lab.js (Henninger et al., 2024). Participants of 18 years or older were recruited through the online platform Sona Systems (n.d.) for students of psychology at the FernUniversität in Hagen. Course credit was offered to all participants. Ethical approval was gained from the departmental ethics committee; the study was conducted in accordance with the ethical standards of the Declaration of Helsinki (2013).

Initially, 130 participants completed the study within an acceptable time frame (without breaks longer than 30 min). As preregistered, we excluded cases of incompletely recorded trials in the experimental task (*n* = 4) or reported severe technical problems (*n* = 1), cases with a total error rate above 25% (*n* = 4), and a participant answering that they could not clearly recognize the viewing direction of the robot avatar (*n* = 1; with a value below 3 on a rating scale from 1 to 5). The final sample size was *n* = 120 (77 female, 43 male, 0 diverse), the age ranging from 18 to 65 years (*M*_age_ = 32.88 years, *SD*_age_ = 11.33 years).

#### *Experimental design*

For the Dot Perspective Task, we applied a 2 × 2 × 2 within-subject design with the factors Perspective (self perspective vs. other perspective), Consistency (consistent vs. inconsistent views between participants and avatars), and Avatar (human avatar vs. robot avatar). The Dot Perspective Task was performed in two successive blocks with either the robot avatar in Block 1 and the human avatar in Block 2 (*n* = 61) or vice versa (*n* = 59). The factor Block order was randomized between participants. While the avatar’s appearance was held constant per block, the factors Perspective and Consistency varied from trial to trial within blocks in a randomized order. The dependent variables were response time and error rate.

#### *Stimuli*

**Fig. 1:**
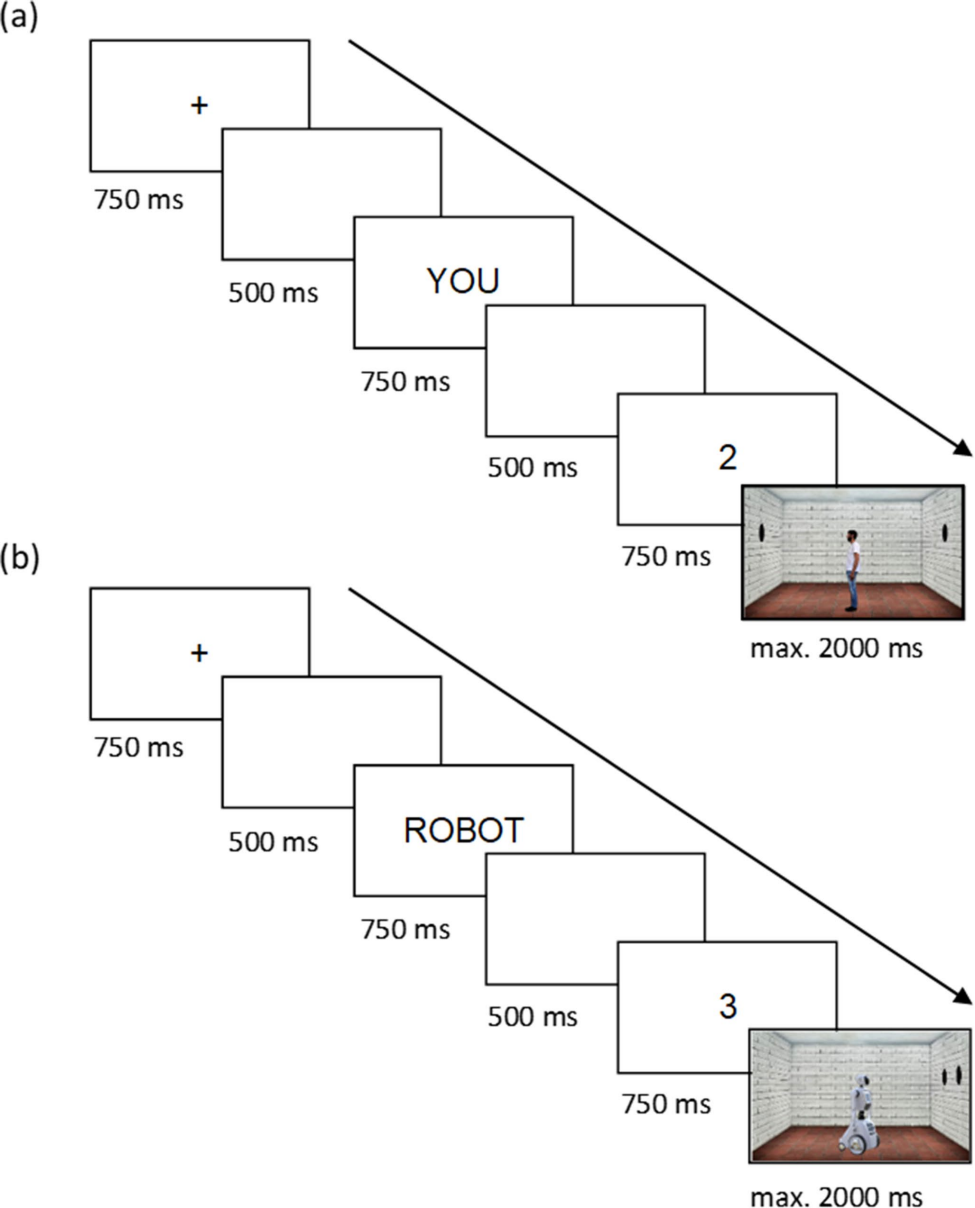
Examples of two trials in the dot perspective task. *(adapted from* Samson et al., 2010). Panel (**a**) shows an inconsistent self perspective trial with a human avatar and a matching answer. The observer, addressed by the pronoun “YOU” sees two dots in the 3D-room, whereas the left-facing male avatar placed in the middle of the room can only see the one dot on the left wall. Since the displayed digit “2” matches the number of dots seen by the participant, the participant should provide a “yes” answer. Panel (**b**) shows a consistent trial with a robot avatar. The right-facing robot avatar, addressed by the word “ROBOT” sees two dots on the right wall. The participants, too, can see these two dots and only these. Since the displayed digit “3” does not match the number of dots seen by the robot avatar (nor by the participant), the participant should indicate a “no” answer. Over the 96 trials (plus 8 filler trials with no dots on the walls) all possible combinations of human vs. robot avatar, self vs. other perspective, consistent vs. inconsistent trials, matching vs. mismatching answer, and right- vs. left-facing avatar were applied

We developed stimuli of a realistic looking male avatar and a robot avatar with low to medium anthropomorphic features, which were both situated in a 3-dimensional room (722 × 433 pixel, see Fig. 1). Avatars were placed in the middle of the room, facing either the left or right wall. Up to three black discs were horizontally aligned on those two walls. The human avatar was based on a photograph of a volunteering human model with neutral clothing (blue jeans and white t-shirt) and no accessories. The robot avatar shared some features with humans (discernable head with clear gaze direction, arm-like limbs on torso), but was overall clearly distinct from a human with, e.g., a box shaped torso on wheels.

#### *Procedure*

Participants gave their informed consent to the experiment and data protection regulations via mouse click, otherwise they could not start the online study. A written overview about the technical requirements and the approximate duration (30 min) was provided. After answering to the demographic questions, participants started the Dot Perspective Task, which began with a detailed instruction and practice trials.

A single trial of the Dot Perspective Task ran as follows (see Fig. 1): A fixation cross was displayed for 750 ms, followed by a blank screen for 500 ms. Next, a prompt with either the word “YOU”, “HE” or “ROBOT” appeared for 750 ms. After another blank page of 500 ms, a single-digit number (“0”, “1”, “2”, or “3”) was shown, finally followed by a 2000 ms presentation of the image of the avatar in the 3-dimensional room. The prompt indicated the perspective that the participant had to adopt (“YOU” in *self perspective* trials, “HE” in *other perspective* trials for the human avatar, “ROBOT” in other perspective trials for the robot avatar). The participant had to compare the number digit to the number of discs “seen” on the walls of the 3D-room image, from the prompted perspective. If the digit matched the number of visible dots participants had to press the right arrow key (“→”), if it did not match, they had to press the left arrow key (“←”). If no key was pressed within 2000 ms, the next trial started automatically. Participants were instructed to keep their left index finger on the left key and their right index finger on the right key during each block. In *consistent trials* the participant and the avatar could see the same number of dots, i.e., all dots were placed on the wall the avatar was facing. In *inconsistent trials*, the number of dots visible to the participant and the avatar differed, i.e., all or some dots were placed on the wall the avatar was not facing.

Before experimental trials, participants performed an initial practice phase with six sample trials of the Dot Perspective Task with robot and human avatars with feedback (“correct”, “wrong”, or “too slow”). The experimental phase consisted of two blocks of 52 trials each, including one block with a robot avatar and one block with a human avatar. No feedback was provided on experimental trials. Four trials in each block were so-called filler trials with 0 dots on each wall. The 48 non-filler trials were balanced with respect to Perspective, Consistency, Matching, and avatar’s gaze direction (right or left).

After the Dot Perspective Task, participants answered control questions on (i) whether they noticed a difference between the avatar’s appearance in the first and the second block, (ii) whether they could clearly see the dots on the walls, and (iii) whether they were able to see the viewing direction of the robot avatar. Next, the perception of the robot was assessed by means of the Godspeed Questionnaire (Bartneck et al., 2009) with the five subscales *anthropomorphism* (5 items, Cronbach’s α = .73), *animacy* (6 items, Cronbach’s α = .74), *likability* (5 items, Cronbach’s α = .90), *perceived intelligence* (5 items, Cronbach’s α = .80), and *perceived safety* (3 items, Cronbach’s α = .79), using 5-point semantic different scales. For additional research questions not reported in this paper we also collected self-report data on participants’ empathy (Paulus, 2009; German version auf the Interpersonal Reactivity Index by Davis, 1983). Finally, a seriousness check and a question on disturbances or potential technical problems during the experiment were administered before participants were fully debriefed about the aim of the study.

#### *Statistical analysis*

Before aggregating data to calculate cell means, the filler trials were excluded. Upper limits for the response times were pre-set by the cut-off of a 2000 ms presentation. In line with Xiao et al. (2022) and Wahn et al. (2025), we only included matching trials in the analysis, and mismatching trials were excluded. The cell means for response times were calculated, including only the trials with correct responses. For each dependent variable, the hypotheses were tested by a 2 (Perspective) × 2 (Consistency) × 2 (Avatar type) within-subject ANOVA. Planned comparisons with paired samples *t*-tests were done to test for consistency effects on fixed levels of Perspective and Avatar type. Statistical analyses were performed in R (data preparation and explorative analyses) and SPSS 29 (IBM SPSS Statistics).

### Results

**Fig. 2:**
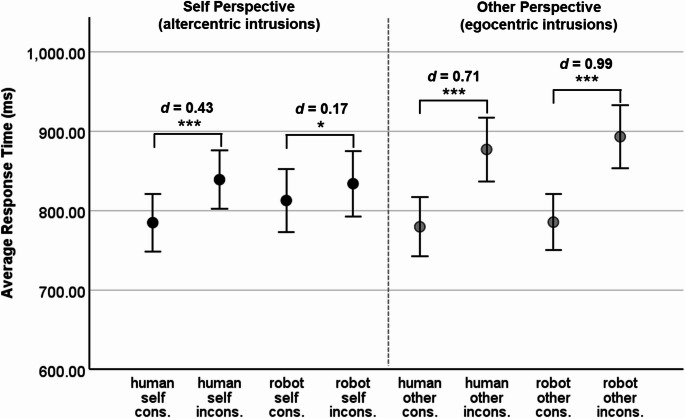
Response Times in Experiment 1 (male human avatar and robot avatar). Note. *N* = 120. Error bars display 95% CIs. * *p* <.05 **, *p* <.01, *** *p* <.001, one-sided t-test. cons. = consistent trials, incons. = inconsistent trials. *d* = Cohen’s *d*

**Fig. 3:**
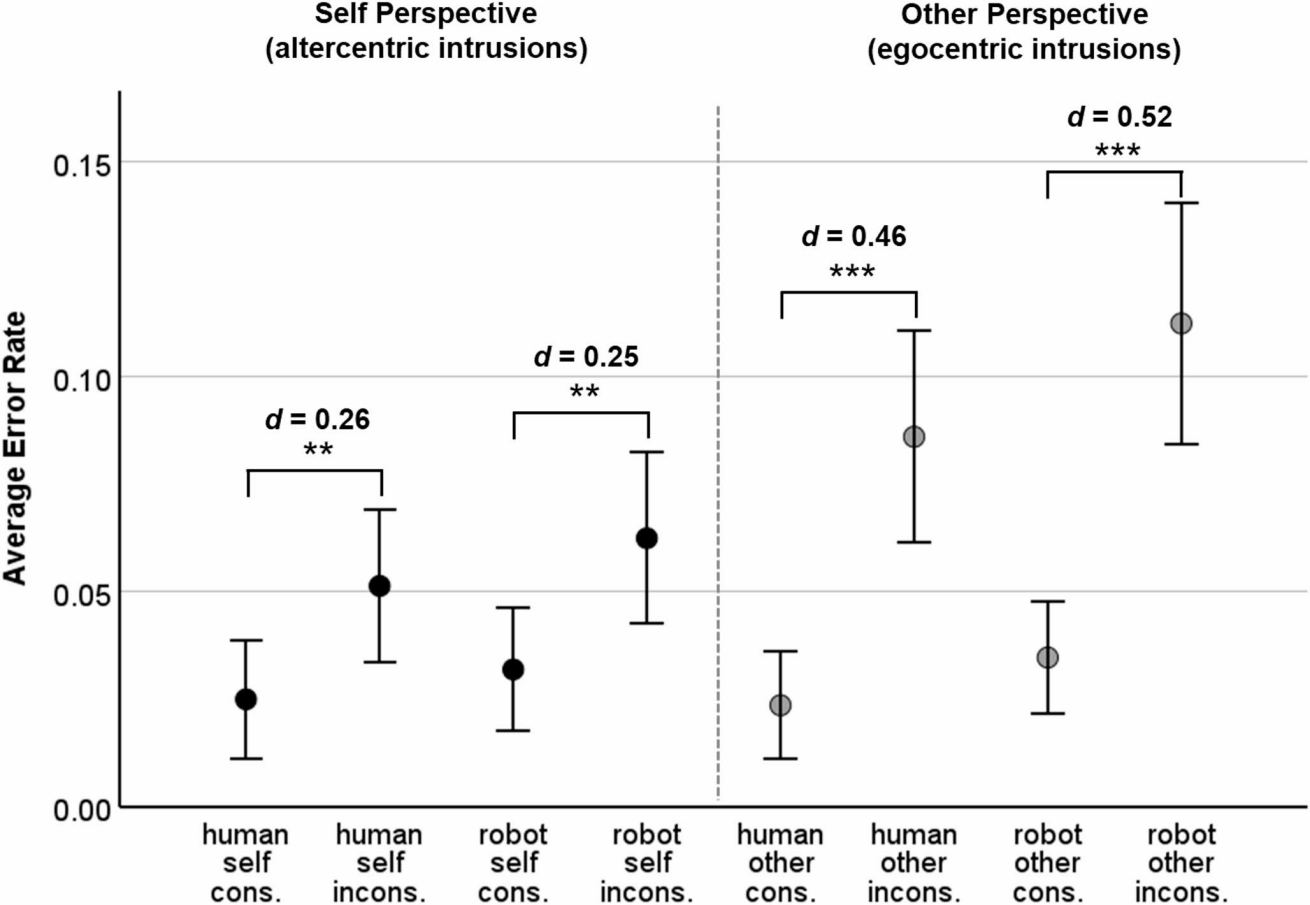
Error Rates in Experiment 1 (male human avatar and robot avatar). Note. *N* = 120. Error bars display 95% CIs. * *p* <.05 **, *p* <.01, *** *p* <.001, one-sided t-test. cons. = consistent trials, incons. = inconsistent trials. *d* = Cohen’s *d*

For the robot avatar, the average ratings on the Godspeed Questionnaire (Bartneck et al., 2009; 5-point semantic different scales from 1 to 5) were as follows: *M*_anthropomorphism_ = 1.73 (*SD* = 0.72), *M*_animacy_ = 2.07 (*SD* = 0.65), *M*_likability_ = 3.18 (*SD* = 0.76), *M*_intelligence_ = 3.30 (*SD* = 0.71), and *M*_safety_ = 2.13 (*SD* = 0.81). Comparing the ratings to those reported in Xiao et al. (2022) showed that the 95% confidence intervals for means overlapped, except for likeability. The average likeability (averaged over experiments 1 and 2) of the robot in Xiao et al. (*M*_likability_ = 2.82, *SD* = 0.72) was lower than the robot’s likability in this study.^2^ Descriptive statistics for response times and error rates are displayed in Figs. 2 and 3, respectively. The main and interaction effects of the two mixed ANOVAs are reported in Table 1.

#### *Response time analysis*

Overall, participants reacted faster to consistent (*M* = 790.85 ms, *SD* = 184.25 ms) than to inconsistent trials (*M* = 861.00 ms, *SD* = 190.77 ms), *F*(1, 119) = 125.57, *p* <.001, η_p_^2^ = .51. The consistency effect was unrelated to the avatars’ appearance (interaction Avatar × Consistency: *F*(1, 119) = 1.01, *p* =. 316, η_p_^2^ < .01). The main effect of Avatar, the Avatar × Perspective interaction, and the Avatar × Perspective × Consistency three-way interaction were not significant (see Table 1). On average, response times were higher in the other perspective (*M* = 834.01 ms, *SD* = 185.74 ms) than in the self perspective (*M* = 817.84 ms, *SD* = 193.73 ms), but the main effect of Perspective did not reach significance, *F*(1, 119) = 3.88, *p* =.051, η_p_^2^ = .03. The highest response times were found for inconsistent trials under the other perspective, the difference between consistent and inconsistent trials being significantly higher under the other perspective than under the self perspective (interaction Perspective × Consistency: *F*(1, 119) = 39.84, *p* <.001, η_p_^2^ = .25). Figure 2 includes the significance levels and effect sizes of pairwise comparisons (one-sided t-tests) of consistent vs. inconsistent trials for each combination of avatar type and perspective: The consistency effect under the other perspective (egocentric intrusion effect) was significant for the human avatar, *t*(119) = 7.82, *p* <.001, *d* = 0.71, as well as for the robot avatar, *t*(119) = 10.81, *p* <.001, *d* = 0.99. The consistency effect under the self perspective (altercentric intrusion effect) was significant for the human avatar, *t*(119) = 4.71, *p* <.001, *d* = 0.43, and also significant for the robot avatar, *t*(119) = 1.83, *p* =.035, *d* = 0.17, yet with a smaller effect size.

#### *Error rate analysis*

Mirroring the response time effects, there was a clear main effect of Consistency, *F*(1, 119) = 53.19, *p* <.001, η_p_^2^ = .31, the error rates for inconsistent trials (*M* = 0.078, *SD* = 0.081) being significantly higher than those for consistent trials (*M* = 0.029, *SD* = 0.041). The Avatar × Consistency interaction was not significant, *F*(1, 119) = 1.14, *p* =.288, η_p_^2^ < .01, and the Avatar × Perspective × Consistency three-way interaction not either (see Table 1). Also, the main effect of Avatar and the Avatar × Consistency interaction were non-significant (see Table 1). Averaged across all other factors, participants made more errors in the other perspective (*M* = 0.064, *SD* = 0.071) than in the self perspective (*M* = 0.043, *SD* = 0.052), as reflected by the main effect of Perspective, *F*(1, 119) = 13.06, *p* <.001, η_p_^2^ = .10. The differences between consistent and inconsistent trials, i.e., the consistency effects, were larger for the other perspective (interaction Perspective × Consistency, *F*(1, 119) = 11.67, *p* <.001, η_p_^2^ = .90). Figure 3 includes the results for pairwise comparisons: The consistency effect under the other perspective (egocentric intrusion effect) was significant and medium-sized for the human avatar, *t*(119) = 5.01, *p* <.001, *d* = 0.46, and for the robot avatar, *t*(119) = 5.64, *p* <.001, *d* = 0.52. The consistency effect under the self perspective (altercentric intrusion effect) was also significant, but smaller in size both for the human avatar, *t*(119) = 2.79, *p* =.003, *d* = 0.26, as well as for the robot avatar, *t*(119) = 2.78, *p* =.003, *d* = 0.25.

#### *Explorative analyses*

On an explorative basis, we tested whether the small, yet significant altercentric intrusions for the robot avatar were affected by perceived human-likeness as assessed through the Godspeed questionnaire (Bartneck et al., 2009). An altercentric intrusion index was operationalized as the difference between average response times for the inconsistent self perspective minus the consistent self perspective robot avatar condition. A multiple regression model with the altercentric intrusion index as the criterion and the five scale means of the Godspeed questionnaire as predictors was not significant, *F*(5, 114) = 2.06, *p* =.08, *R*^2^_adj_ = .04. The predictor likability was significant with a negative estimate (*b* = − 42.69, *t* = − 2.34, *p* =.027). Anthropomorphism (*b* = 33.77, *t* = 1.88, *p* =.063), animacy (*b* = 17.31, *t* = 0.79, *p* =.431), perceived intelligence (*b* = 13.30, *t* = 0.67, *p* =.501), and perceived safety (*b* = − 22.92, *t* = − 1.53, *p* =.129) did not significantly predict the altercentric intrusion index.

Additionally, we tested whether the altercentric intrusions for the robot avatar were affected by the specific characteristics of the within-subjects design. E.g., in terms of contrast and priming effects, the altercentric intrusions of the robot avatar might have been different for participants who had seen the robot avatar without prior exposure to another type of avatar and participants who had worked on the Dot Perspective Task with a human avatar in the block before encountering the robot avatar. A t-test for independent samples with presentation order as the independent variable and the altercentric intrusion index as the dependent variable showed no significant effect, *t*(117.24) = 0.27, *p* =.078, *d* = 0.05. This finding was also supported by non-significant ANOVA interactions: For an overall exploration of block and sequential effects, we extended the above reported three-factorial ANOVA models to four-factorial ANOVA models, adding the factor Block (block 1 vs. block 2). For both dependent variables, response time and error rate, a 2 (Perspective) × 2 (Consistency) × 2 (Avatar type) × 2 (Block) within-subject ANOVA was conducted. Strong main effects of Block revealed lower response times in Block 2 compared to Block 1, *F*(1, 118) = 87.09, *p* <.001, η_p_^2^ = .42, and respectively lower error rates, *F*(1, 118) = 26.93, *p* <.001, η_p_^2^ = .19. For the response times, the Perspective × Block interaction was significant, too, *F*(1, 118) = 13.43, *p* <.001, η_p_^2^ = .25, implying that the effect of reacting faster in Block 2 than in Block 1 was more pronounced for the other perspective than for the self perspective. All other interactions including the factor Block in the two ANOVAs were non-significant (all *p* values ≥ 0.28). Thus, the result patterns regarding avatar type and (altercentric) intrusions were not affected by the sequential order.

**Table 1:** Results of the within-subjects ANOVAs in experiment 1 for response times and error rates in the Dot perspective task

	*F*	df	*p*	η_*p*_^2^
Response times				
Avatar	0.90	1, 119	.346	< .01
Perspective	3.88	1, 119	.051	.03
** Consistency**	**125.57**	**1**,** 119**	**< .001**	**.51**
Avatar × Perspective	< 0.01	1, 119	.970	< .01
Avatar × Consistency	1.01	1, 119	.316	< .01
** Perspective × Consistency**	**39.84**	**1**,** 119**	**< .001**	**.25**
Avatar × Perspective × Consistency	3.61	1, 119	.060	.03
Error rates				
Avatar	3.58	1, 119	.061	.03
** Perspective**	**13.06**	**1**,** 119**	**< .001**	**.10**
** Consistency**	**53.19**	**1**,** 119**	**< .001**	**.31**
Avatar × Perspective	0.58	1, 119	.447	< .01
Avatar × Consistency	1.14	1, 119	.288	< .01
** Perspective × Consistency**	**11.67**	**1**,** 119**	**< .001**	**0.90**
Avatar × Perspective × Consistency	0.22	1, 119	.642	< .01

*N* = 120. Perspective (self vs. other), consistency (consistent vs. inconsistent trials), and avatar type (human vs. robot) were manipulated within subjects. Significant effects are highlighted in bold

### Discussion

An overall advantage of processing consistent trials was observed: irrespective of the perspective and the avatar, consistent trials were answered faster and with greater accuracy than inconsistent trials, supporting the assumption that distinguishing the self vs. other perspective, i.e., perspective selection, requires executive function resources to inhibit the non-relevant, yet automatically activated perspective (Qureshi et al., 2010; Gardner & Thorn, 2025). For the other perspective, this consistency effect on response time and error rate was remarkably stronger (*d* ≥ 0.46) than for the self perspective (*d* ≥ 0.17), which is in line with earlier studies showing that the egocentric biases exceed altercentric biases (e.g., Samson et al., 2010).

Inconsistent with our hypotheses, the avatar’s appearance did neither moderate the degree of altercentric nor egocentric intrusions. The sequential effects of encountering a human avatar prior to a robot avatar on the magnitude of altercentric intrusions were tested, but could be ruled out. We predicted that – as in Xiao et al. (2022, Exp. 2) – especially the altercentric intrusions (as an indicator of automatic L1-VPT) would be stronger in the human avatar condition than in the robot avatar condition, but the levels of intrusions did not differ significantly between the two avatar types. In Experiment 1, for both human and robot avatars, significant egocentric and altercentric intrusions were observed. Whether the altercentric intrusions for the robot avatar could be predicted by the Godspeed dimensions on perceived human-likeness remained inconclusive, with only one out of five dimensions being a significant predictor.

## Experiment 2

Given that most participants in Experiment 1 were female, it was conceivable that the female majority of the sample perceived both the robot avatar and the male human prototype as too different from themselves to engage in similarity-induced automatic L1-VPT. In order to test if the findings from Experiment 1 could be generalized to a greater variety of avatars, Experiment 2, also involving a predominantly female sample, used a female prototype instead of a male avatar, the rest of the procedure being equivalent do Experiment 1.

### Method

#### *Sample size rationale and sample*

Paralleling Experiment 1, as preregistered (https://osf.io/dszb7) we aimed for a total of 130 participants who completed the online study without breaks longer than 30 min. This number was reached after a data collection period from 8 November 2023 to 16 November 2023. Recruitment via Sona systems (n.d.) and course credit incentives were as in Experiment 1. Ethical approval was gained from the departmental ethics committee; the study was conducted in accordance with the ethical standards of the Declaration of Helsinki (2013). Records were excluded in case of technical problems with the experimental software (*n* = 2), total error rates above 25% (*n* = 7) or an error rate of 100% for least one ANOVA cell (*n* = 2), denying serious participation (*n* = 1), answering ‘no’ to the control questions about whether the experimental block 1 and 2 showed different avatars (*n* = 4) and whether one could see the viewing direction of the robot avatar (*n* = 1; with a value below 3 on a rating scale from 1 to 5). The final sample size was *n* = 113 (88 female, 24 male, 1 diverse), the age ranging from 18 to 67 years (*M*_age_ = 31.52 years, *SD*_age_ = 10.38 years).

#### *Experimental design, stimuli, and procedure*

As in Experiment 1, a 2 × 2 × 2 within-subject design with the factors Perspective (self perspective vs. other perspective), Consistency (consistent vs. inconsistent views between participants and avatars), and Avatar (human avatar vs. robot avatar) was applied. For the robot avatar condition, the same avatar picture as in Experiment 1 was used, whereas the human avatar was modified. In Experiment 2, the human avatar depicted a young female person in blue jeans and a white t-shirt. Changing the human avatar’s sex from male (Experiment 1) to female (Experiment 2) was the only difference between the two studies. As an adjustment to the Dot Perspective Task, the prompt for other-perspective trials with human avatar was now “SHE” (instead of “HE” in Experiment 1). The presentation duration for trials and their subcomponents as well as the number of trials per block (52 trials including 4 filler trials without dots) were as in Experiment 1. Based on a randomized procedure, participants worked on the Dot Perspective Task either with the robot avatar in Block 1 and the human avatar in Block 2 (*n* = 57) or vice versa (*n* = 58). For the Godspeed Questionnaire on robot assessment (Bartneck et al., 2009), the data of Experiment 2 showed that the internal consistencies of the five subscales were comparable to Experiment 1 (*anthropomorphism*: Cronbach’s α = .72; *animacy*: Cronbach’s α = .79; *likability*: Cronbach’s α = .93; *perceived intelligence*: Cronbach’s α = .83; *perceived safety*: Cronbach’s α = .85).

#### *Statistical analysis*

The criteria for trial-wise exclusions, the calculation of cell means, and ANOVAs were the same as in Experiment 1.

### Results

**Fig. 4:**
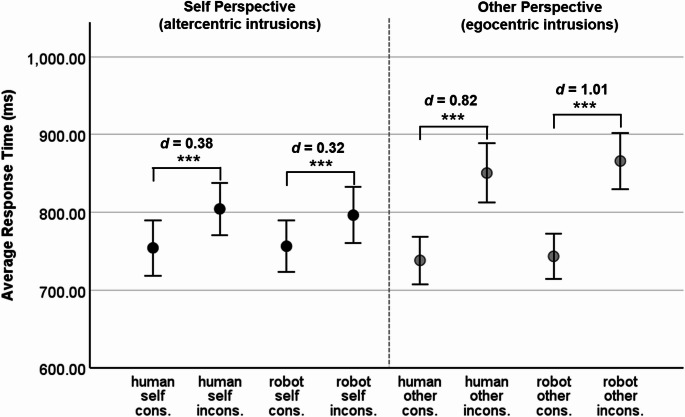
Response Times in Experiment 2 (female human avatar and robot avatar). Note. *N* = 113. Error bars display 95% CIs. * *p* <.05 **, *p* <.01, *** *p* <.001, one-sided t-test. cons. = consistent trials, incons. = inconsistent trials. *d* = Cohen’s *d*

**Fig. 5:**
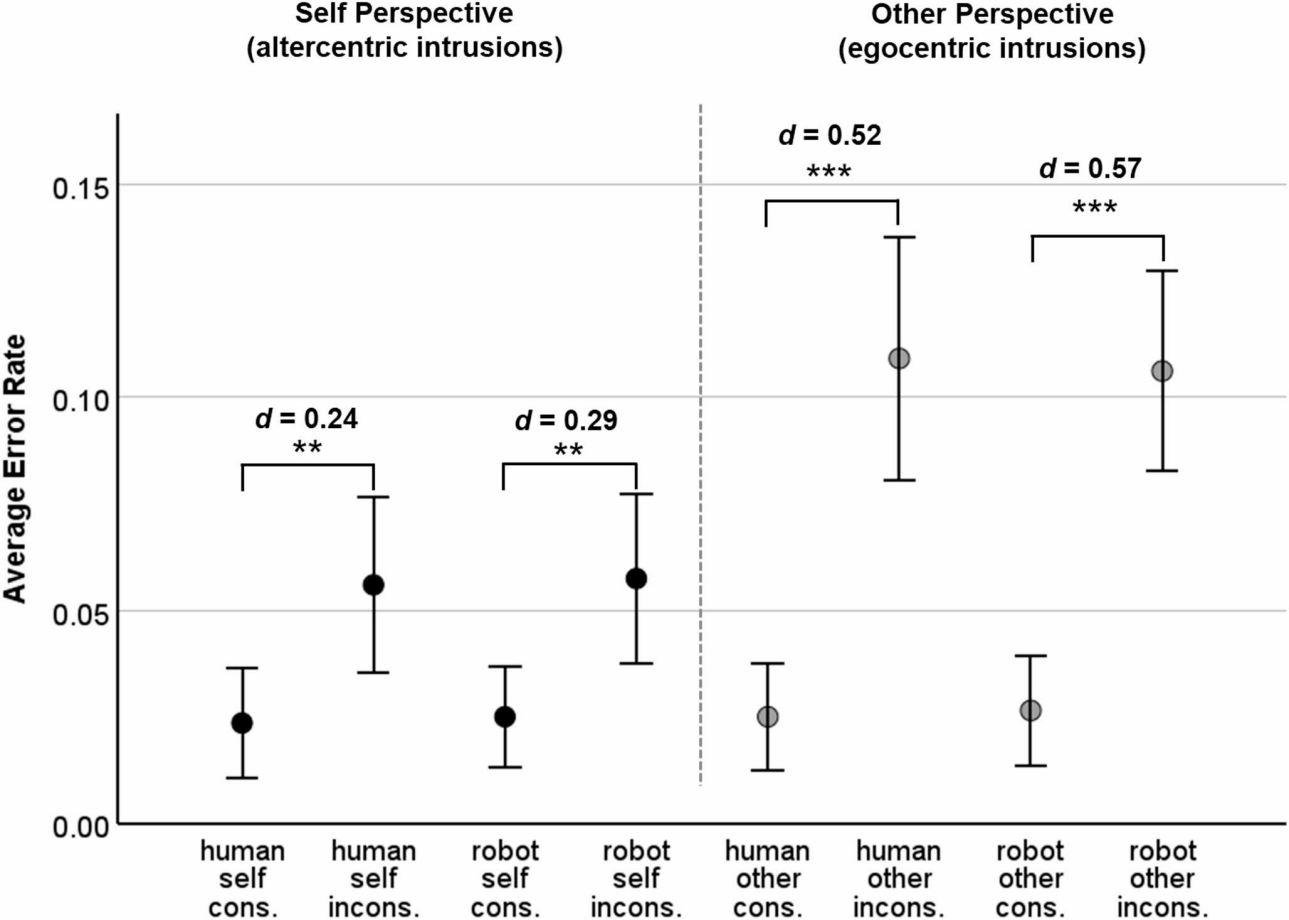
Error Rate in Experiment 2 (female human avatar and robot avatar). Note. *N* = 113. Error bars display 95% CIs. * *p* <.05 **, *p* <.01, *** *p* <.001, one-sided t-test. cons. = consistent trials, incons. = inconsistent trials. *d* = Cohen’s *d*

For the robot avatar the average ratings on the Godspeed Questionnaire (Bartneck et al., 2009; 5-point semantic different scales from 1 to 5) were as follows: *M*_anthropomorphism_ = 1.62 (*SD* = 0.62), *M*_animacy_ = 2.03 (*SD* = 0.69), *M*_likability_ = 3.14 (*SD* = 0.81), *M*_intelligence_ = 3.40 (*SD* = 0.69), and *M*_safety_ = 2.08 (*SD* = 0.89). Comparing these ratings to the scale-transformed ratings from Xiao et al. (2022), we found that the average ratings for perceived intelligence of the robot in Xiao et al. was lower (*M*_intelligence_ = 3.04, *SD* = 0.79). For the other mean ratings, the 95% confidence intervals for means overlapped. The descriptive statistics for response times and error rates are displayed in Figs. 4 and 5. The main effects and interactions of the corresponding within-subjects ANOVAs are reported in Table 2.

**Table 2:** Results of the within-subjects ANOVA in experiment 2 for (a) response times and (b) error rates in the Dot perspective task

	*F*	df	*p*	η_*p*_^2^
Response times				
Avatar	0.12	1, 112	.734	< .01
** Perspective**	**9.05**	**1**,** 112**	**.003**	**.08**
** Consistency**	**192.66**	**1**,** 112**	**< .001**	**.63**
Avatar × Perspective	1.31	1, 112	.255	.01
Avatar × Consistency	< 0.01	1, 112	.995	< .01
** Perspective × Consistency**	**26.63**	**1**,** 112**	**< .001**	**.19**
Avatar × Perspective × Consistency	0.83	1, 112	.365	< .01
Error rates				
Avatar	< 0.01	1, 112	.945	< .01
** Perspective**	**14.59**	**1**,** 112**	**< .001**	**.12**
** Consistency**	**71.21**	**1**,** 112**	**< .001**	**.39**
Avatar × Perspective	0.36	1, 112	.851	< .01
Avatar × Consistency	0.04	1, 112	.837	< .01
** Perspective × Consistency**	**12.25**	**1**,** 112**	**< .001**	**.10**
Avatar × Perspective × Consistency	0.03	1, 112	.868	< .01

*N* = 113. Perspective (self vs. other), consistency (consistent vs. inconsistent trials), and avatar type (human vs. robot) were manipulated within subjects. Significant effects are highlighted in bold

#### *Response time analysis*

As in Experiment 1, participants reacted faster to consistent (*M* = 747.96 ms, *SD* = 151.74 ms) than to inconsistent trials (*M* = 829.24 ms, *SD* = 163.57 ms), *F*(1, 112) = 192.66, *p* <.001, η_p_^2^ = .63. This consistency effect was unrelated to the avatar’s appearance (interaction Avatar × Consistency: *F*(1, 112) < 0.01, *p* =.995, η_p_^2^ < .01). The main effect of Avatar, the Avatar × Perspective interaction, and the Avatar × Perspective × Consistency three-way interaction were not significant (see Table 2). As in Experiment 1, the response times were higher in the other perspective (*M* = 799.44 ms, *SD* = 154.16 ms) than in the self perspective (*M* = 777.78 ms, *SD* = 164.36 ms); in Experiment 2, this main effect of Perspective was significant, *F*(1, 112) = 9.05, *p* =.003, η_p_^2^ = .08. The highest response times were observed for inconsistent trials under the other perspective, the difference between consistent and inconsistent trials being significantly higher under the other perspective than under the self perspective (interaction Perspective × Consistency, *F*(1, 112) = 26.63, *p* <.001, η_p_^2^ = .19). Figure 4 displays the results of pairwise comparisons of consistent vs. inconsistent trials for each combination of avatar type and perspective. The consistency effect under the other perspective (egocentric intrusion effect) was significant for the human avatar, *t*(112) = 8.70, *p* <.001, *d* = 0.82, as well as for the robot avatar, *t*(112) = 11.63, *p* <.001, *d* = 1.01. The consistency effect under the self perspective (altercentric intrusion effect) was somewhat smaller than the egocentric intrusion effect, yet highly significant both for the human avatar, *t*(112) = 3.99, *p* <.001, *d* = 0.38, and for the robot avatar, *t*(112) = 3.44, *p* <.001, *d* = 0.32.

#### *Error rate analysis*

Paralleling the response time effects, the 3-factorial ANOVA on error rates also yielded a main effect of Consistency, *F*(1, 112) = 71.21, *p* <.001, η_p_^2^ = .39, with lower error rates in consistent (*M* = 0.025, *SD* = 0.036) than in inconsistent trials (*M* = 0.082, *SD* = 0.076). The Avatar × Consistency interaction was not significant, *F*(1, 112) = 0.04, *p* =.837, η_p_^2^ < .01, and the Avatar × Perspective × Consistency three-way interaction not either (see Table 2). Also, there was no significant main effect of Avatar and no significant Avatar × Perspective interaction (see Table 2). Overall, with a main effect of Perspective, the error rate in the other perspective (*M* = 0.067, *SD* = 0.066) was significantly higher than in the self perspective (*M* = 0.041, *SD* = 0.052), *F*(1, 112) = 14.59, *p* <.001, η_p_^2^ = .12. The differences between consistent and inconsistent trials were larger for the other perspective than for the self perspective (interaction Perspective × Consistency, *F*(1, 112) = 12.25, *p* <.001, η_p_^2^ = .10). In Fig. 5 the results for pairwise comparisons are displayed: The consistency effects under the other perspective (egocentric intrusions) were significant both for the human, *t*(112) = 5.51, *p* <.001, *d* = 0.52, and for the robot avatar, *t*(112) = 6.00, *p* <.001, *d* = 0.57. The consistency effects under the self perspective (altercentric intrusions) were smaller, yet significant for the human, *t*(112) = 2.59, *p* =.005, *d* = 0.24, and for the robot avatar, *t*(112) = 3.17, *p* =.001, *d* = 0.29.

#### *Explorative analyses*

Exploring potential effects of the robot avatars’ perceived human-likeness on altercentric intrusions, we calculated a multiple linear regression with the reaction-time based altercentric intrusion index as the criterion and the five scale means of the Godspeed questionnaire as predictors. Neither the total model, *F*(5, 107) = 1.33, *p* =.258, *R*^2^_adj_ = .01, nor the single predictors were significant: anthropomorphism (*b* = − 40.03, *t* = − 1.74, *p* =.086), animacy (*b* = 38.64, *t* = 1.69, *p* =.094), likability (*b* = 10.09, *t* = 0.54, *p* =.588), perceived intelligence (*b* = − 0.10, *t* = − 0.01, *p* =.996), perceived safety (*b* = 19.74, *t* = 1.24, *p* =.183).

As in Experiment 1 we also investigated if the presentation order, i.e., having seen a human avatar in a Dot Perspective Task block before the robot avatar or not, had an effect on the robot avatar’s altercentric intrusions. This was not the case, *t*(96.74) = 0.77, *p* =.444, *d* = 0.14. For an overall exploration of block and sequential effects we conducted two additional 2 (Perspective) × 2 (Consistency) × 2 (Avatar type) × 2 (Block) within-subject ANOVAs. The strong main effects of Block showed lower response times, *F*(1, 111) = 96.99, *p* <.001, η_p_^2^ = .47, and lower error rates, *F*(1, 111) = 6.01, *p* =.016, η_p_^2^ = .05, in Block 2 than in Block 1. For the response times, the Perspective × Block interaction, *F*(1, 111) = 15.24, *p* <.001, η_p_^2^ = .12, as well as the Consistency × Block interaction, *F*(1, 111) = 6.11, *p* =.015, η_p_^2^ = .05, were significant, reflecting that the effect of reacting faster in Block 2 than in Block 1 was (a) stronger for the other perspective than for the self perspective and (b) stronger for the inconsistent than for the consistent trials. Most importantly as to the avatar-specific intrusions, all other interactions including the factor Block were non-significant (all *p* values ≥ .151). Therefore, the result patterns regarding avatar type and (altercentric) intrusions were not affected by the sequential order.

### Discussion

Experiment 2 complemented Experiment 1 by replicating the main results on level 1 visual perspective taking with a female, instead of a male, human avatar in the Dot Perspective Task. Even more clearly than in Experiment 1, the main hypothesis that the human avatar would trigger (automatic) level 1 visual perspective taking to a greater extent than a robot avatar was not confirmed. Significant egocentric intrusions, i.e., consistency effects for the other perspective, and – more importantly – altercentric intrusions, i.e., consistency effects for the self perspective, were observed for both human and robot avatars. The magnitude of intrusions was not moderated by avatar type, contrary to our expectations. Again, moderating effects of presentation order on the magnitude of altercentric intrusions could be ruled out.

In contrast, the amplitude of intrusions was moderated by perspective. As in Experiment 1, for both dependent measures (response times and error rates) there were strong egocentric intrusions and significant, but less pronounced altercentric intrusions. The longest response times and the highest error rates were observed when the other perspective had to be taken and when the information under this perspective conflicted with the participant’s own perspective. The altercentric intrusions for the robot avatar could not be predicted by perceived human-likeness of the robot avatar.

## General discussion

When seeing (non-humanoid) robot avatars, do we – automatically – adopt their visual perspective to the same extent as we would do with other human beings? Following the empirical results from Xiao et al. (2022) and the theoretical mentalizing approach (e.g., Frith & Frith, 2012; Ramsey et al., 2013; Samson et al., 2010), we predicted that visual perspective taking should occur more strongly for the human avatars than for the robot avatars, especially for altercentric intrusions. However, both Experiment 1, using an online variant of the Dot Perspective Task with a male avatar, and Experiment 2, using the same paradigm with a female avatar, did not confirm this hypothesis. A robot avatar (i.e., a non-humanoid robot) did not trigger level 1 visual perspective taking to a lower extent than a human avatar did, a finding we observed in both experiments. The significant altercentric intrusions remained stable when controlling for sequential order effects; i.e., the perception of the avatar type presented in the first block of the task (e.g., that a human avatar really looked human) did not affect the perception of the avatar type presented in the second block (e.g., that the robot avatar looked machinelike as a contrast to the human avatar presented before).

Given that altercentric intrusions were detectable for both dependent measures (response times and error rates) and for the two avatar types, robots as well as male and female humans, we can conclude that the perspective of the avatar at least evoked interference: Even though observers were instructed to apply their own perspective, they did not fully ignore the avatars’ perspective or orientation, but responded in a prolonged manner and made more errors when the two perspectives conflicted with one another. Altercentric intrusions as such are in line with two opposing theoretical approaches: According to the *mentalizing* approach the intrusions reflect that people implicitly adopt another agent’s perspective because of the agent’s or the context’s social nature (Frith & Frith, 2012; Samson et al., 2010). In contrast, according to the *submentalizing* approach (Heyes, 2014; Santiesteban et al., 2014), the intrusions would reflect domain-general attentional processes such as that the avatar’s gaze direction makes the observer focus on that part of the visual scene which represents the avatar’s perspective (e.g., for a left-facing avatar paying attention to the dots on the left wall only instead of both walls simultaneously). Hence, the fact that both avatar types yielded comparable levels of altercentric intrusions could on the one hand mean that the consistency effects for both, human and robotic, avatars were caused by non-social, domain-general attentional cues, in line with the submentalizing approach. On the other hand, it could mean that both avatars were perceived as social, triggering the processes of mentalizing also for a non-humanoid robotic avatar. Data from the Godspeed questionnaire (Bartneck et al., 2009) show that the non-humanoid avatar used in Experiments 1 and 2 was rated low on the animacy scale and very low on the anthropomorphism scale, comparable to the ratings of the robots in Xiao et al. (2022). Since no control ratings were collected for the human avatars, we cannot say for sure that the robot avatar was indeed perceived lower on anthropomorphism than the human, yet the values close to the lower bound of the scale make it unlikely that participants fully anthropomorphized the robot. As exploratory analysis showed, anthropomorphism and animacy ratings did not predict the extent of altercentric intrusions, either. Yet, for strict control and replication of the partly inconclusive regression models reported above, we recommend that future studies assess anthropomorphism ratings for all used avatar types and also a measure of perceived similarity between the participant and the avatar (e.g., Inclusion of Other in the Self (IOS) Scale; Shafaei et al., 2020). In terms of model simplicity, the mentalizing account, requiring that each observer has to process anthropomorphism and to attribute human characteristics to the non-humanoid robot by default, is less parsimonious than the submentalizing account; Occam’s razor would imply to favor the simpler explanation. Thus, the non-social, domain-general attentional mechanisms offer a parsimonious explanation for the finding of altercentric intrusions and L1-VPT in the Dot Perspective Task.

Paradigms aiming to contribute to the ongoing debate about mentalizing vs. submentalizing in level 1 visual perspective taking include, among others: (a) replacing avatars by non-social objects with directional cues, e.g., arrows (Santiesteban et al., 2014), (b) using scenes in which gaze direction alone does not cue the correct answer (e.g., some dots in the avatar’s gaze direction are hidden behind obstacles; Santiesteban et al., 2014), (c) employing belief manipulations, e.g., avatars with goggles which are supposed to be either transparent or intransparent, so that in spite of facing a wall the avatar cannot see the dots (e.g., Furlanetto et al., 2016). All of these – so far primarily used with human avatars – could also be applied to robot avatars in order to disentangle attention-based and socially-grounded processes in visual perspective taking of robot avatars (see also Wahn et al., 2025).

Based on our results from two studies with non-students (Exp. 1) and German students from a distance learning university (Exp. 1 and 2) we could not replicate the findings by Xiao et al. (2022) from a sample of Chinese university students who were younger than our participants. Differences in age and cultural background might be a possible explanation, but this would rather predict the opposite pattern of results: Assuming that young Asian students show higher engagement in human-robot interaction (for a comparison between China, Korea, and Germany see Li et al., 2010), participants from Xiao et al. (2022) might have anthropomorphized the robot avatar more than our participants, making altercentric intrusions for robot avatars more likely (and not less likely). Our original assumption that the chosen design of the machine-like looking robot would entail even lower perceived human-likeness than the humanoid robot in Xiao et al. (2022) was not supported by the data. Indeed, there was a tendency to perceive the robot in our study as more likable (Experiment 1) and more intelligent (Experiment 2). Yet the comparable levels of perceived anthropomorphism and animacy can rule out the possibility that the altercentric intrusion effects for the robot avatar in our study were due to a higher perceived human-likeness of the robot. However, caution is warranted as we compare here two studies conducted on different populations and using slightly different scale formats. Against this background, our data provides no support for the account of similarity-induced mentalizing in level-1 visual perspective taking, as found in earlier studies (e.g., Ferguson et al., 2018). Theoretical assumptions of models on self-other integration in joint action involving two acting individuals (Dolk et al., 2014; Hommel et al., 2001; Prinz, 1997) may not be transferable to visual perspective taking with static pictures of avatars not producing any response (events).

In our study, the robot avatar was chosen to be representative of a machine-like robot with some human-like features (discernable head with clear gaze direction, arm-like limbs on torso). Unlike every day robots such as NAO (for an overview see Robaczewski et al., 2021), prior experience and familiarity were unlikely to have confounding effects on perspective taking. Yet future research could also address this question by controlling for prior experience, which in turn might be related to age and cultural background (Goldman & Poulin-Dubois, 2024; Kim & Kim, 2013). This would allow insights regarding the mere appearance hypothesis (Zhao & Malle, 2022), which posits that humanlike appearance makes people adopt an avatar’s perspective, but not the knowledge-based attribution of human characteristics. Wahn and colleagues (Wahn & Berio, 2023; Wahn & Wiese, 2025) have recently challenged the mere appearance hypothesis by demonstrating that technical objects without human appearance can also trigger visual perspective taking, provided that the devices (cameras) imply the social presence of a human observer. Hence, varying both the appearance of robotic avatars and whether a robot implies human presence could provide insights into what extent perspective taking is driven by visual features and by socially grounded attributions or by an interaction of both.

The strongest and most robust effect in both Experiment 1 and Experiment 2 was not the significant altercentric intrusion effect, but the significant, large-sized egocentric intrusion effect. The finding that the level of egocentric intrusions exceeds that of altercentric intrusions is not new (see Samson et al., 2010) and in line with Xiao et al. (2022)’s findings. It can be interpreted as a self-dominance effect. Being highly available and prone to automatic activation, information from the self-perspective is processed first (Bradford et al., 2015) and thus needs more cognitive effort to be suppressed than information from the other perspective. Interestingly, as long as the perspective of the observer and the avatar were consistent, response times and error rates were not significantly higher for the other perspective than for the self perspective. Instead, the remarkably larger consistency effects for the other perspective, i.e., the egocentric intrusion effect, appeared due to the prolonged responses and increased error rates for inconsistent other perspective trials. Hence, based on the data at hand, adopting another agent’s perspective did not necessarily require enhanced processing costs per se, but only did so when combined with a conflict of information, i.e. when self and other perspective provide conflicting information (cf. Heyes, 2014; Rubio-Fernandez et al., 2022). This contributes to the yet unresolved debate regarding the relative effort or automaticity of perspective selection and perspective calculation in level 1 visual perspective taking (see e.g., Qureshi & Monk, 2018; Todd et al., 2017). Our results would be in line with an adaptive information processing mechanism such that under high (perceptual) load, observers readily ignore the information about the cued perspective as soon as they realize that this information does not affect the answer in a consistent trial, reflecting an early mechanism of information reduction (Lavie & Dalton, 2014). Only in a second step, in inconsistent trials, observers invest the additional cognitive effort to process the cued perspective in more depth.

In terms of cognitive load, an important methodological consideration is the selection of the experimental task paradigm. In our study, as in Experiment 2 by Xiao et al. (2022), we applied a so-called *explicit tracking* procedure in which participants are randomly cued before each trial to take either the avatar’s or their own perspective. The alternative procedure is no explicit tracking, where participants are instructed at the beginning of a block to adopt a specific perspective (e.g., to respond from the avatar’s perspective throughout the entire block; see Xiao et al., 2022, Experiment 1). As demonstrated by Holland et al. (2021), the experimental task design influences the effect of perspective consistency; the explicit tracking paradigm makes the factor ‘perspective’ more salient and adds additional cognitive load to the task in terms of switch costs. Switch costs (Ferguson et al., 2017) and working memory load (He et al., 2024; Qureshi & Monk, 2018) due to the required internal updates of the cued perspective in each trial can overlay the consistency effects in a distorting manner due to the additional cognitive load.

Further limitations include the general methodological concerns for online experiments, the settings being not as well standardized as in the laboratory (participation required either a laptop or a computer with an external monitor, but the screen sizes varied between participants and disturbances during the Dot Perspective Task could occur), the fact that the human avatar images were, though made of photographs of real humans, only static in nature, and the overall criticism which is put forward against the Dot Perspective Task as an indicator of level 1 visual perspective taking (Rubio-Fernandez et al., 2022). To address these points in future research and to go a few steps further, lab experiments with a physical human robot interaction and examining level 1 as well as level 2 perspective tasks with mental rotation would be an interesting option. Sticking to virtual avatars, which are relevant in digital communication and media, one could test further the hypothesis of similarity-fostered perspective taking by integrating realistic (3D) photographs of the avatars into the Dot Perspective Task. Such technical extensions were not possible for the experiments reported here, but should be feasible with improved technologies in the near future, yielding the maximum perceived similarity between observer and avatar with the maximum likelihood to induce conflicts of self-other discrimination.

Another question of relevance concerns the stability of perspective taking processes over time. An exploratory analysis showed that in both experiments, participants reacted faster and were more accurate in the second block of the task as compared to the first block. For the response times, this presumed effect of short-term learning and familiarization with the task was more pronounced under the other perspective than under the self perspective (in both experiments) and for inconsistent trials than for consistent trials (in Experiment 2), suggesting that within a short time frame already people could train their executive functions (cf. Liepelt et al., 2011; Wang et al., 2020), learning to handle the cognitive and executive demands arising from information conflicting with their own perspective: They were at least partially able to overcome an initial preference for (a) the self perspective over the other perspective and (b) the consistent over the inconsistent perspectives (see Bukowski & Samson, 2017). A question would be whether this short-term effect turns out to persist in a longitudinal study. Similar to task switching and multitasking (Liepelt et al., 2011; Strobach et al., 2012) it might be possible to train performance in the Dot Perspective Task up to a certain degree so that effects of cognitive and executive noise factors (e.g., effortfully retrieving which keypress represents a yes or no answers) diminish, making the true effects of perspective consistency and avatar type stand out more clearly. This in turn would tackle the not yet fully solved theoretical question about the relative contributions of attentional and socially-grounded processes in visual perspective taking and the practical implication of whether human-like design can help to support human-robot-interaction by means of facilitated perspective taking.

## Data Availability

The aggregated data is available at: https://osf.io/a89bt. The study material can be found online here: https://osf.io/g5um2.

## Footnotes

2 Due to a different scale format the Godspeed ratings were not directly comparable to the metrics by Xiao et al. (2022).While Xiao et al. used 9-point semantic differential scales, we applied the 5-point semantic differential scales as reported in the original study of Bartneck et al. (2009). For the comparison reported here, we transformed the average ratings from Xiao et al. (Experiment 1 and 2 combined) to a 5-point scale format.

## Declarations

### Public significance statement

The success of human-robot-interaction depends on how well people can anticipate a robot’s behavior, which also implies adopting the robot’s point of view. This research starts with the assumption that people can adopt the perspective of a (social) human being more easily than that of a (not so social) robot. Finding full-fledged perspective taking for both, human and robot avatars in a well-established experimental task with static images of avatars, we conclude that submentalizing represents a valid explanation for visual perspective taking. We critically discuss the method and its potential confounding factors (e.g., working memory load) and conclude that more realistic settings are desirable for future research on visual perspective taking.
